# Optometry student clinical practice at public health facilities: A systematic review

**DOI:** 10.4102/hsag.v29i0.2441

**Published:** 2024-03-22

**Authors:** Raserogole F. Segooa, Vanessa R. Moodley

**Affiliations:** 1 Department of Optometry, College of Health Sciences, University of KwaZulu-Natal, Durban, South Africa

**Keywords:** clinical supervision, optometry, student clinical practice standards, student clinical training, student clinical training facilities

## Abstract

**Background:**

Clinical training, supervision and practice are the most important aspects of health profession education, including optometry. Institutions implore various methods for students to gain access, exposure and experience in different clinical environments, away from their normal academic settings.

**Aim:**

This review aimed to investigate studies and related documentary evidence to determine existing standards and methods for educational institutions in conducting optometry clinical training at the external sites.

**Setting:**

The electronic databases – ProQuest One, Scopus, EBSCOhost, Sabinet, Science Direct and Google Scholar – were searched systematically for studies on the implementation of workplace clinical training of undergraduate optometry students.

**Methods:**

The study followed the Joanna Briggs Institute (JBI) systematic review methodology and a systematic search of various electronic databases was conducted for studies on implementation of workplace clinical training. Of the 450 full-text studies searched, 13 studies were found to be reputable sources of evidence and were included in this systematic review.

**Results:**

Four themes relating to student clinical training emerged, namely, clinical training approaches implemented, expected minimum standards at the training sites, clinical training environment wherein students and supervisors find themselves and clinical competence of the supervisors and students. They encompass important factors to consider in the planning and provision of quality, efficient and effective student clinical supervision at the external training facilities.

**Conclusion:**

There is a dearth of scholarly studies to guide clinical training of optometry training within the public health sector. However, more studies are undertaken in other health disciplines, and they provide generic guidelines, which can be adapted for optometry.

**Contribution:**

The article highlights the need for further studies in optometry student clinical training, focussing on programme designs and standardisation of clinical training in multi-institutional, low-income contexts.

## Introduction

Clinical education and training, supervision by experienced clinicians and exposure to patients presenting with a diverse range of anomalies or diseases are fundamental for students in health professions education. Clinical training provides students an opportunity to transfer theoretical knowledge gained within academic settings into real-life practice settings Pashmdarfard and Shafarood (2018:114), fostering exposure and experience in different clinical settings, away from their normal academic environment. Seaman, Green and Freire (2022:5364) describe clinical education and training as a fundamental placement of students at clinical, health or organisational settings for a period during their educational training, be it rural, urban, private and/or public health settings. There are different approaches to clinical training and practice that may vary according to disciplines, each applying their specific clinical skills requirements when implementing student training (Pashmdarfard & Shafarood 2018:119). Clinical training requires best practice policy and design to support the workforce (Seaman et al. 2022:5383).

Optometry is historically known for conducting urban-based, private practice-oriented clinical training under the supervision of private optometrists (Ebrahim et al. 2019:1). This strategy may have significantly contributed to the overwhelming majority of graduates directly entering private sector practices on completion of their degrees. It is only in recent years that optometry introduced rural and/or community or public sector-based clinical training under the supervision of public sector optometrists (Ebrahim et al. 2019:2). This review aimed to investigate studies and related documentary evidence to determine existing standards and methods for educational institutions in conducting optometry clinical training at the external sites. It focussed on the availability of clinical training standards and protocols, to guide public sector-based optometry training. The research question used to guide the review was, ‘What documentary resources are available to guide all aspects of undergraduate optometry student’s clinical training, at the external training facilities?’

## Research methods and design

A qualitative systematic review was conducted to systematically search and bring together research evidence on a topic from various studies and draw the findings (Seers 2015:36). A preliminary search for reviews in-progress or completed was conducted on PROSPERO database for systematic reviews in May 2022, and no systematic reviews on the topic were found. This review followed the Joanna Briggs Institute (JBI) systematic review methodology (Page et al. 2021b:160), and its protocol was registered with PROSPERO before conducting the study (registration number CRD42022330909).

### Search strategy

The electronic databases – ProQuest One, Scopus, EBSCOhost, Sabinet, Science Direct and Google Scholar – were searched systematically for studies on the implementation of workplace clinical training of undergraduate optometry students. Searches were conducted from May 2022 to July 2022. The search strategy was tailored to meet the requirements of each database by using a combination of keywords related to optometry student clinical training or supervision and clinical practice. For example, keywords: ‘optometry’ AND ‘student’ AND ‘clinical supervision’ AND ‘clinical practice’ were used for ProQuest One search.

### Inclusion and exclusion criteria

The review considered studies that reported on implementation of clinical training at the external training sites or workplace (Table 1), taking into consideration content, quality, perception, impact, process and/or delivery of student clinical training, practice or supervision. The pool for optometry studies was very limited, leading reviewers in extending the search to include other health professions.

**TABLE 1:** Inclusion and exclusion criteria.

Inclusion criteria	Exclusion criteria
Full-text studies published in English from 2017 to 2022.	Studies not reporting on implementation of student clinical training and/or supervision.
Studies related to the implementation of optometry student clinical training and/or supervision.	
Studies on workplace clinical training and/or supervision in all health professions.	

### Selection of the studies

Guided by the inclusion and exclusion criteria (Table 1), two reviewers independently conducted the searches extracting resources by title, abstract and full text. The reviewers discussed and compared the papers included at every stage to determine their accordance and discrepancies. Where they differed, reviewers engaged in further discussion, until they reached consensus.

### Search findings

The database search yielded 450 studies and examination by title yielded 74 studies, then 72 studies after removal of duplicates (Figure 2). Examination of potential studies by abstract yielded 33 studies and examination of full texts of the remainder of the studies yielded 21 studies. The 12 studies excluded based on full-text examination were found to not be related to student clinical training and/or supervision and practice. The 21 articles were re-examined, and after thorough review, 7 articles were not in accordance with the aims and objectives of the study and were excluded. A total of 14 eligible studies were critically appraised in order to determine their level of evidence and quality.

**FIGURE 1:**
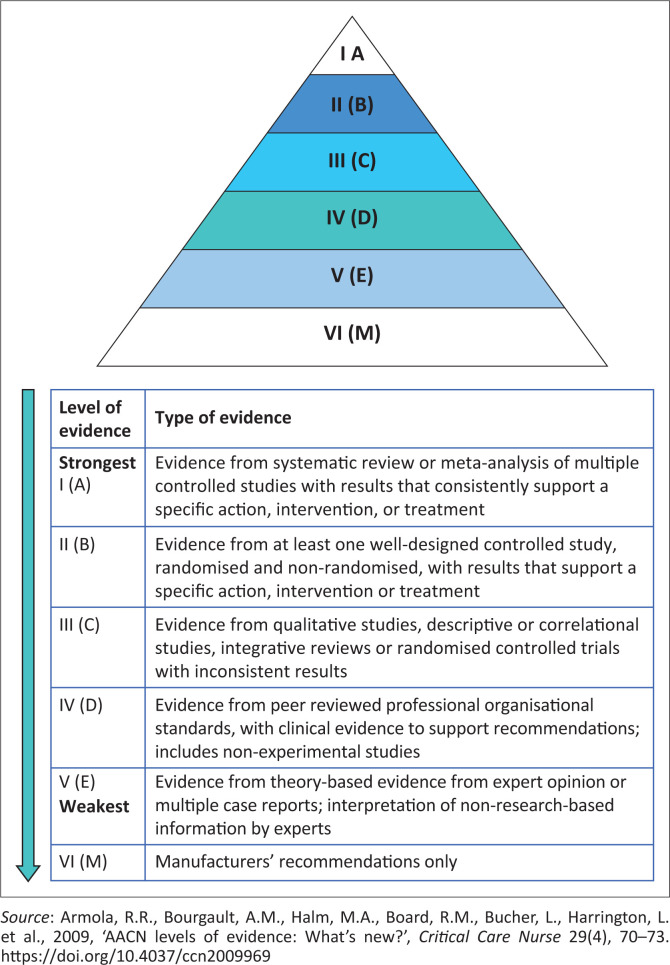
American Association Critical Care Nurse’s evidence-levelling system.

**FIGURE 2:**
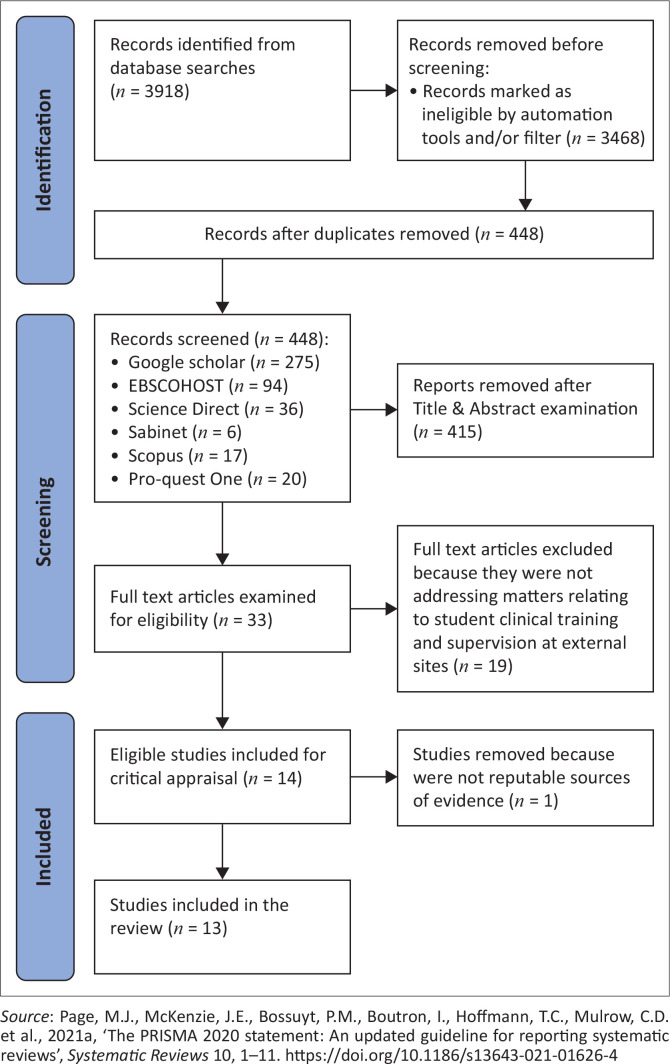
Prisma flow diagram of the selection process.

Different critical appraisal tools were used to assess the methodological quality of 14 eligible studies (Appendix 1). The level of evidence and quality of studies were graded according to the American Association Critical Care Nurses (AACN) evidence levelling system (Figure 1) (Armola et al. 2009:70–73). Eleven studies were appraised according to the JBI critical appraisal checklists (Whiting et al. 2003:1–3); of which, four were appraised according to JBI systematic reviews checklist (Aromataris et al. 2015:132–140), four according to qualitative studies checklist (Lockwood, Munn & Porritt 2015:179–187), one according to experimental studies checklist (Tufanaru et al. 2020:3–10) and two studies according to text and opinion (descriptive) checklist (McArthur et al. 2015:188–195). Mixed Methods Appraisal Tool (MMAT) sourced from Hong et al. (2018a:285–291) and Hong et al. (2018b:1–11) was used to appraise one study, and two other studies were appraised according to the Non-Research Literature Appraisal Tool (Yale New Haven Health 2016:1–3). Out of 14 studies, 13 studies were found to be reputable sources of evidence (Figure 2) and were included in this systematic review (Table 2).

**TABLE 2:** Qualifications and/or professions involved in clinical supervision included in the review.

Discipline	Number of studies
Optometry	4
Ophthalmology	2
Optician and/or Dispensing optician	1
Pharmacy	1
Pharmacy Technician	1
Allied (optometry, dietetics and nutrition, occupational therapy and physiotherapy)	1
Non-medical professions (nursing, pharmacy, midwifery, optometry, podiatry, chiropractic, orthotics and prosthetics, physiotherapy, speech therapy, audiology, social work, oral health, medical radiation science, dietetics and nutrition, psychology, paramedics occupational therapy and other)	1
Rehabilitation Science (occupational therapy, physical therapy, optometry, auditory, speech therapy and technical orthopaedics)	1
Medical and Allied (audiology, biokinetics, exercise and leisure sciences, dentistry, occupational therapy, optometry, pharmaceutical sciences, physiotherapy and speech language pathology)	1
TOTAL	13

Based on the level of evidence (Figure 1), the reviewers agreed to grade the 13 studies that were found to be reputable sources of evidence according to a methodological quality grading of: low quality for studies that scored below 40%; good or medium quality for those that scored 40% to 80% and high quality above 80%. In all, 12 studies were found to be of high quality, scoring above 80%, whereas one study was found to be of good or medium quality, scoring 80%. Most studies scored 90% and above; however, some studies that were graded to be of high and medium quality did not meet all critical appraisal criteria against which they were measured (Appendix 1).

### Data extraction and synthesis

Thirteen studies were found to be potential resources to guide clinical training and supervision of undergraduate optometry student training, outside the training institutions (Table 2). Eight studies discussed external clinical training in optometry, four of which were purely about optometry, whereas the other four discussed various disciplines, including optometry. Five studies discussed external clinical training in other health professions.

**FIGURE 3:**
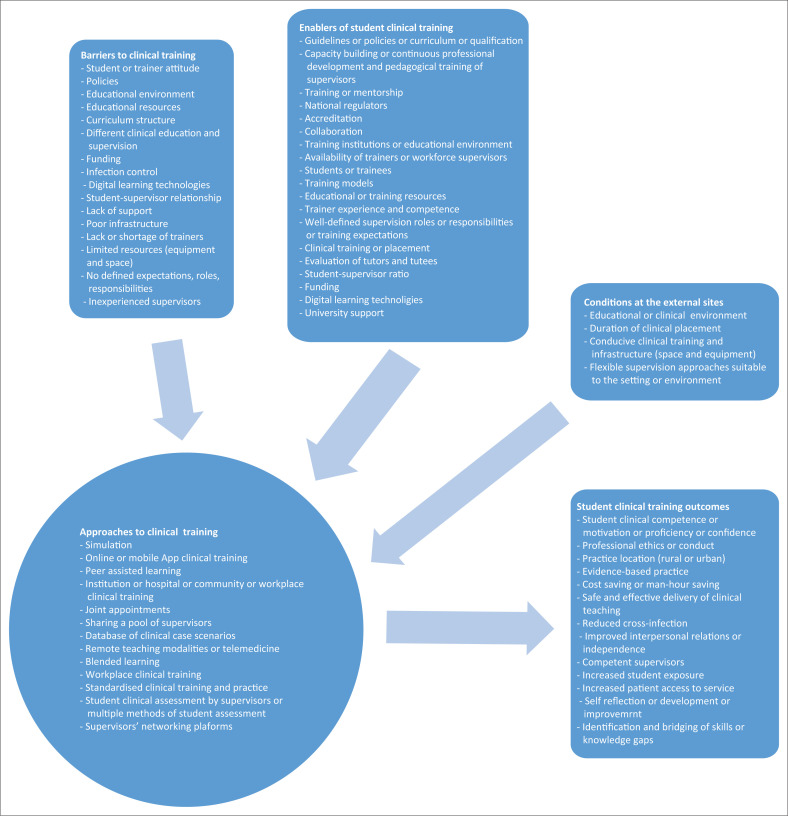
Summary of current study codes.

A standardised tool was developed to extract relevant information from the selected studies, and the extracted data are summarised in a table format (Appendix 2). A qualitative analysis was conducted, whereby texts from the summarised studies’ results were extracted and systematically transformed into highly organised and concise summary of key results (Erlingsson & Brysiewicz 2019:94), to describe a specific phenomenon (Downe-Wambolt in Bengtsson 2016:9). Data were then condensed and labelled to formulate codes (Figure 3), whose patterns were compared and organised into categories leading to themes. That assisted this review in identifying examples of student clinical supervision systems or approaches, enablers, barriers and outcomes as shown in Figure 3. This process required several reiteration to validate data before establishing themes and subthemes emanating from data (Bradshaw, Atkinson & Doody 2017:5).

## Results

From the data extracted, four themes emerged, namely clinical training approach, expected minimum standards, clinical training environment and clinical competence.

### Theme 1: Clinical training approach

Educational and clinical supervision are the two key elements for effective supervision, and they have complementary roles that offer distinct but connected contributions to student learning and support (Hindi et al. 2022:7). Clinical supervision support learners in developing their proficiency and confidence, and in translating the knowledge and skills gained, into clinical practice, whereas educational supervision support learners in navigating the clinical pathways (Hindi et al. 2022:7). Both are mainly conducted by qualified professionals (Hindi et al. 2022:7), but there are other clinical training approaches where senior students can supervise junior students in a clinical setting, namely peer-assisted learning (PAL) (Van Vuuren 2017:10). According to Boud in Van Vuuren (2017:9), PAL is a two-way reciprocal learning activity involving the sharing of knowledge, and it is a vehicle to help undergraduate healthcare students learn how to teach (Van Vuuren 2017:9). Peer-assisted learning has been implemented effectively in nursing and medicine; it is fairly new in the allied health professions (Van Vuuren 2017:9).

Coronavirus disease 2019 (COVID-19) has proven that e-learning is possible and moreover essential; it shifted physical teaching to online teaching and in doing so reformed education in general (Jonuscheit et al. 2021:154–155). Some training centres introduced pooling of resources and implemented joint appointments (dual academia-clinician appointments), while others introduced a variety of web-based online platforms, such as simulation, dry and wet labs, virtual reality and e-learning (Chee et al. 2021:262; Dean et al. 2020:1077–1078; Jonuscheit et al. 2021:152; Lee et al. 2020:1737–1738). Simulation was found to be of benefit during clinical training because trainees displayed confidence when they are not practising on real patients, significantly improving their performance (Flanagan & De Souza 2018:428). Tele-medicine and/or consultation, on the other hand, have emerged and been adopted by most practitioners as the preferred mode of providing care in hospital and private practice settings, as well as during clinical skills training (Sehgal et al. 2021:962). Remote supervision, like tele-consultation, allows students to be placed at the external facilities to expose them to other health skills and practice opportunities, while simultaneously addressing staff shortage at those facilities (Seaman et al. 2022:5363).

### Theme 2: Expected minimum standards

In sub-Saharan Africa, there is a move for the national regulator to oversee training of ophthalmologists, the examination standards and accreditation of training centres, for programmes to be implemented successfully (Dean et al. 2020:1067–1073). This is enabled by policies, guidelines and standards being available for the countries and institutions to use during training (Dean et al. 2020:1079–1080). Although there is a system in place to standardise ophthalmology training in the sub-Saharan countries, there is, however, a general shortage of ophthalmologists at accredited district facilities used as the training centres, resulting in a shortage of trainers (Dean et al. 2020:1074; Flanagan & De Souza 2018:432–433). Staff shortage is not only encountered in ophthalmology, but other professions do have similar problems, including optometry. Authors, Dean et al. (2020:1077) and Jonuscheit et al. (2021:152) agree and suggest that to manage a shortage of trainers and not disadvantage trainees, institutions should explore other training modes through the development of broader collaborative training programmes with external or international stakeholders.

Irrespective of the facility or sector, it is important that clinical training of professionals at workplaces is structured and delivered in a suitable, equitable and transparent manner, with roles, responsibilities and competencies of trainers and trainees clearly defined (Schafheutle, Jee & Willis 2018:1025–1026). Those responsible for clinical placements should prepare and provide both students and supervisors with clear guidance covering detailed training expectations (Kirkman et al. 2022:9). Moodley and Singh (2020:30) emphasise the importance for academics to ensure students meet the required competencies through community-based clinical training assessments. Numerous queries arose related to assessments traditionally conducted within the formal academic setting; including appropriateness of methods for community-based settings and capacity of clinical supervisors (Moodley & Singh 2020:30–32). In such instances, designing assessments for external settings is a challenge as the learning environments are not standardised, and students would be assessed by a number of tutors, with varied levels of academic skills, making clinical assessments difficult to control (Moodley & Singh 2020:33).

It is also important for the universities to implement student support systems for students to access help before relationships with supervisors are strained, as most students highlighted a lack of support from the university, resulting in a degree of anxiety (Kirkman et al. 2022:9). Student training can offer facilities an increase in human resource compliment and reduced patient waiting period for health services, and high patient numbers at public sector facilities offer students an opportunity to access large patient numbers and a wide range of clinical cases, which is in line with the regulatory authorities (Ebrahim et al. 2019:3–4).

### Theme 3: Clinical training environment

Individual factors (student and trainer characteristics), environmental factors (policies, curriculum) and clinical factors (trainer experience and competence, clinical surrounding and resources, training style), should be identified and planned for as they affect student clinical education, and if not well considered, the expected quality of training may be affected negatively (Pashmdarfard & Shafarood 2018:120). If the clinical training environment is poor, with deficiencies such as a lack of educational or training centres, poor patient numbers and limited resources (funding, trainers, space and/or equipment), trainers may feel overburdened and reluctant to train, ultimately affecting the clinical competence of their assigned students (Dean et al. 2020:1079–1080; Ebrahim et al. 2019:5–6; Pashmdarfard & Shafarood 2018:118–119). This can have both a positive and negative effect on student practice preference post-qualification (Seaman et al. 2022:5381–5382). Under-resourced environments, may on a positive note, as seen with COVID-19, serve as opportunities for institutions and training facilities to explore alternative modes of training that are cost-effective, contextually appropriate, accessible, offer standardised training and improve patient safety through introduction of stricter hygiene practices (Flanagan & De Souza 2018:429; Lee et al. 2020:1754–1755; Sehgal et al. 2021:962).

In a study by Kirkman (2022:9–10), students reported that, they experienced times when relationships with supervisors were strained, resulting in them being anxious, without university support. Student and supervisor characteristics (individual factors) should be taken into consideration during planning and implementation stages of clinical training as they can affect the outcome (Pashmdarfard & Shafarood 2018:120). It is important that supervision models are flexible and adaptable to the settings, learner needs and the healthcare workforce skills (Hindi et al. 2022:7).

Lack of clarity with respect to the roles, responsibilities and accountability of trainees placed at private sector external sites was found to limit their exposure to a wide range of activities during training and that made them feel isolated (Schafheutle et al. 2018:1025). Their counterparts at the public sector sites were found to have had more benefits because they were allocated with other trainees for peer support, qualified professionals as role models, senior staff as supervisors, support staff and other health professionals and thus, not confined to one area, but interacted with a variety of professionals from different disciplines (Schafheutle et al. 2018:1025). There is, however, no conclusive evidence on whether shorter or longer placement periods positively or negatively impact upon the preferred practice location, post-qualification (Seaman et al. 2022:5382).

### Theme 4: Clinical competence

To maximise student clinical competence, the gap between theoretical knowledge and clinical practice should be minimised (Pashmdarfard & Shafarood 2018:119). Educators must have appropriate clinical qualifications, be competent in clinical practice and have the required experience, as they play a critical role in ensuring that students are competent (Pashmdarfard & Shafarood 2018:119). It is important for universities to be aware of levels of supervisor competence and experience to be able to allocate them or place students accordingly; this is supported by Ebrahim et al. (2019:6), who found junior optometrists to be knowledgeable in theory, but lacking clinical experience, requiring further maturity to serve as clinical teachers. On the other hand, senior optometrists have the necessary experience and maturity to train students, but lack adequate knowledge on current theoretical aspects (Ebrahim et al. 2019:5). Experienced trainers could help facilitate the professional identity development of trainees (Hindi et al. 2022:8).

Training the trainer programmes were found to equip the trainers in teaching, and cascading knowledge and skills to trainees (Dean et al. 2020:1073); however, the transfer and assessment of desirable character, attitude, ethics and responsibilities are yet to be proven (Dean et al. 2020:1078). Peer-assisted learning, ongoing professional development and pedagogical training of supervisors, including assessment methods, are recommended in order to keep supervisors informed and up-to-date with new developments that current students are being taught (Kirkman et al. 2022:9–10; Moodley & Singh 2020:32; Van Vuuren 2017:10–11). This is important, as most of the students participating in community-based education programmes are exit-level students, needing to meet graduate competencies in preparation for the work environment (Moodley & Singh 2020:32).

## Implications and recommendations

The reviewed studies were found to be relevant resources to guide clinical training and supervision of undergraduate optometry students, outside the training institutions. Where possible, the external facilities should implement more than one student clinical training approach to complement each other and align with the recommended regulatory and university requirements. Approaches using the qualified professionals (educational and clinical supervision) and/or students (PAL) as supervisors could be adapted to support optometry clinical training at facilities external to the universities, as they were proven effective. The former have complementary roles that offer distinct, but connected contributions to student learning and support (Hindi et al. 2022:7), whereas the latter is a reciprocal learning activity involving the sharing of knowledge from senior to junior healthcare students, and a vehicle to help senior undergraduate students learn how to teach (Van Vuuren 2017:9). Peer-assisted learning can be used in clinical optometry training to assist students who could not grasp a concept as presented by the qualified professionals, who might find it easy to engage the senior students for better understanding.

Web-based online platforms are effective; however, they would require the facilities to invest in technology infrastructure, its maintenance and training the trainers, to ensure that these platforms are sustainable. For example, simulation was found to enhance the confidence and performance of trainees as they are not practising on real patients (Flanagan & De Souza 2018:428); tele-medicine and/or consultation, on the other hand, allow students to be placed in external facilities to expose them to other health skills and practice opportunities, while simultaneously addressing staff shortage at the facilities (Seaman et al. 2022:5363). Blended learning would therefore be ideal for clinical education going forward.

To ensure that the expected minimum standards of optometry are met and are uniform among the universities offering the programme, it is recommended that the national regulators oversee the training, the examination standards and accreditation of training centres, which would be enabled by policies, guidelines and standards available to use, for successful implementation (Dean et al. 2020:1067–1073). Although there is a system in place to standardise training of health professions, there is a general shortage of trainers at most accredited district facilities used as the training centres, (Dean et al. 2020:1074; Flanagan & De Souza 2018:432–433). Optometry programmes could benefit from resource pooling and joint appointments, to address trainer shortages, save costs for the training institutions and ensure that curricula are harmonised and as a result improve the quality of clinical training.

Authors do agree that there is a need for clinical assessments to be conducted at the external sites; however, supervisors need to be trained first as learning environments are not standardised and students would be assessed by a number of tutors, with varied levels of academic skills (Moodley & Singh 2020:33). Goal setting and definite objectives should be determined prior placements (Robertson in Naidoo 2006:3), as ill-defined roles can complicate student-supervisor relationship. Universities should implement student support systems, to intervene when there are misunderstandings between students and supervisors, before the relationships are strained (Kirkman et al. 2022:9).

Good management and harmonious working relationship lead to a desirable working environment and learning experience (Laurent & Weidner 2002:251). It is important for all those involved in the planning, facilitation and implementation of clinical training programme at the external facilities, including optometry, to interact frequently and have the course objectives outlined, to ensure alignment and uniform clinical training outcomes in all sectors. Optometry programmes should consider incorporating the individual, clinical and environmental factors when planning for student clinical placements and ensure that the working environment is conducive for student learning, as poor environment can negatively affect student training. It is therefore incumbent on the facilities and the universities to identify the threats towards clinical training and take advantage of the opportunities at hand, in order to bridge the gaps and improve the environment.

Positive environmental factors can influence the quality of clinical placements, as well as the intentions and attitudes of students, including student preference to practice in those areas, post-qualification. Successful implementation of student training was found to have dual benefits; it can offer facilities an increase in human resource complement with reduced patient waiting period for health services and also offer students an opportunity to access large patient numbers and a wide range of clinical cases, which increases their competence level (Ebrahim et al. 2019:3–4). In order to produce competent students, trainers must be informed, with a variety of expertise, be receptive to students and be interested in new techniques (Laurent & Weidner 2002:251). Furthermore, the selected sites should have high staff morale, sound inter-disciplinary patient management, with supervisors who understand students and can facilitate the learning process through sound teaching skills (Laurent & Weidner 2002:251). To maximise student clinical competence, it may be beneficial for optometry programmes to pair junior and senior optometrists conducting student training, to support and complement each other, as a result, optimise the student learning experience.

Limitations of this review include limited information on optometry clinical training in general, and on clinical training at the public health facilities. However, studies on the clinical training and supervision in other health professions were available and they were included in this study. The highlight of the review is the discovery of the clinical training approaches and standards, which can be adapted to suit optometry programmes and ensure effective clinical training at the public health facilities.

## Conclusion

There are documentary resources available to guide all aspects of undergraduate optometry student clinical training, at the external training facilities. Clinical training and supervision should be well planned, with the required standards outlined and clear objectives for all stakeholders. The training facilities should have resource capacity and be conducive for student clinical training with competent supervisors, knowledgeable on recent clinical developments. Universities should continuously empower clinical supervisors through standardisation workshops and other continuous development activities to ensure confidence in teaching and mentoring of trainees. Students must have ongoing channels of communication and support.

More studies in optometry should be conducted to review student clinical training, with emphasis on the standardisation of outcome competencies. As evident from the review, student clinical training experience at various facility settings influence practice decisions post-qualification. It is therefore recommended that all universities fully implement the public sector placements, in order to promote the return of students, to service the underprivileged and underserved communities.

## Appendix 1

### Critical appraisal tool report of eligible *descriptive studies*.

Predetermined questions:

Is the source of the opinion clearly identified?

Does the source of opinion have understanding in the field of expertise?

Are the interests of the relevant population the central focus of the opinion?

Is the stated position the result of an analytical process, and is there logic in the opinion expressed?

Is there reference to the extant literature?

Is any incongruence with the literature/sources logically defended?

**Table:**

Study type, author, year and title	Q1	Q2	Q3	Q4	Q5	Q6
Descriptive study by Jonuscheit et al. (2020):	Y	Y	Y	Y	Y	Y
COVID-19: Ensuring safe clinical teaching at university optometry schools						
Survey by Sehgal et al. (2021):	Y	Y	Y	Y	Y	Y
Impact of COVID-19 on Indian Optometrist: a student, educator and practitioner’s perspective						

Y, yes.

**Table:**

Category	Study 1	Study 2
Study design level of evidence	III (C)	III (C)
Quality grading	6/6 yes = 100% ≥ A – High quality	6/6 yes = 100% ≥ A – High quality
Is this a reputable source of evidence?	Yes	Yes

### Critical appraisal tool report of eligible *literature review studies*.

Predetermined questions:

Is the subject matter under review clearly stated?

Is relevant timely literature included (most sources within 5 years of seminal work)?

Is there a meaningful analysis of the conclusions in the literature?

Are gaps in the literature identified?

Are recommendations for practice clear?

**Table:**

Study type, author, year and title	Q1	Q2	Q3	Q4	Q5
Literature review study by Van Vuuren (2017):	Y	N	Y	Y	Y
Integrated literature review of undergraduate peer teaching in allied health professions					

Y, yes; N, no.

Study design level of evidence: I (A)

Quality Grading: 4/5 yes = 80% ≥ A – High quality

Is this a reputable source of evidence? Yes X No ☐

### Critical Appraisal Tool report of eligible *article review studies*.

The predetermined questions:

Was the aim of the project clearly stated?

Was the method adequately described?

Were processes or outcomes measure identified?

Were results adequately described?

Was interpretation clear and appropriate?

Are components of cost/benefit analysis described?

**Table:**

Study type, author, year and title	Q1	Q2	Q3	Q4	Q5	Q6
Article review study by Flanagan and Desouza (2018):	N	N	N	Y	Y	Y
Simulation in ophthalmic training						

Y, yes; N, no.

Study design level of evidence: IV (D)

Quality Grading: 3/6 Yes = 50% ≥ B – Good quality with major flaws, thus Low quality

Is this a reputable source of evidence? Yes ☐ No X

### Critical appraisal tool report of eligible *experimental studies*.

Predetermined questions:

Was true randomisation used for assignment of participants to treatment groups?

Was allocation to treatment groups concealed?

Were treatment groups similar at the baseline?

Were participants blind to treatment assignment?

Were those delivering treatment blind to treatment assignment?

Were outcomes assessors blind to treatment assignment?

Were treatment groups treated identically other than the intervention of interest?

Was follow-up complete and if not, were differences between groups in terms of their follow-up adequately described and analysed?

Were participants analysed in the groups to which they were randomised?

Were outcomes measured in the same way for treatment groups?

Were outcomes measured in a reliable way?

Was appropriate statistical analysis used?

Was the trial design appropriate, and any deviations from the standard RCT design (individual randomisation, parallel groups) accounted for in the conduct and analysis of the trial?

**Table:**

Study type, author, year and title	Q1	Q2	Q3	Q4	Q5	Q6	Q7	Q8	Q9	Q10	Q11	Q12	Q13
Experimental study by Chee et al. (2021):	Y	Y	Y	Y	U	U	Y	Y	Y	Y	Y	Y	Y
Assessing the use of smartphone app to teach eye screening to opticians													

Y, yes; U, unclear.

Study design level of evidence: I (A); Quality Grading: 11/13 yes = 85% ≥ A – High quality; Is this a reputable source of evidence? Yes X No ☐

### Critical appraisal tool report of eligible *mixed methods studies*.

Predetermined questions:

QUALITATIVE

Is the qualitative approach appropriate to answer the research question?

Are the qualitative data collection methods adequate to address the research question?

Are the findings adequately derived from the data?

Is the interpretation of results sufficiently substantiated by data?

Is there coherence between qualitative data sources, collection, analysis and interpretation?

QUANTITATIVE DESCRIPTIVE

Is the sampling strategy relevant to address the research question?

Is the sample representative of the target population?

Are the measurements appropriate?

Is the risk of nonresponse bias low?

Is the statistical analysis appropriate to answer the research question?

MIXED METHODS

Is there an adequate rationale for using a mixed methods design to address the research question?

Are the different components of the study effectively integrated to answer the research question?

Are the outputs of the integration of qualitative and quantitative components adequately addressed?

Are the divergences and inconsistencies between quantitative and qualitative results adequately addressed?

Do different components of the study adhere to the quality criteria of each tradition of the methods involved?

**Table:**

Study type, author, year and title	Q1.1	Q1.2	Q1.3	Q1.4	Q1.5	Q4.1	Q4.2	Q4.3	Q4.4	Q4.5	Q5.1	Q5.2	Q5.3	Q5.4	Q5.5
Mixed methods study by Schafheutle et al. (2018):	Y	Y	Y	Y	Y	Y	CT	Y	N	Y	Y	Y	Y	Y	Y
The influence of learning environment on trainee pharmacy technicians’ education and training experience															

Y, yes; N, no; CT, cannot tell.

Study design level of evidence: III (C)

Quality Grading: 13/15 Yes = 87% ≥ A – High quality

Is this a reputable source of evidence? Yes X No ☐

### Critical appraisal tool report of eligible *qualitative studies*.

Predetermined questions:

Is there congruity between the stated philosophical perspective and the research methodology?

Is there congruity between the research methodology and the research question or objectives?

Is there congruity between the research methodology and the methods used to collect data?

Is there congruity between the research methodology and the representation and analysis of data?

Is there congruity between the research methodology and the interpretation of data?

Is there a statement locating the researcher culturally or theoretically?

Is the influence of the researcher on the research and vice versa addressed?

Are the participants and their voices adequately represented?

Is the research ethical according to current criteria or for recent studies, and is there evidence of ethical approval by an appropriate body?

Do the conclusions drawn in the research report flow from the analysis or interpretation of the data?

**Table:**

Study type, author, year and title	Q1	Q2	Q3	Q4	Q5	Q6	Q7	Q8	Q9	Q10
Qualitative study by Kirkman (2022):	Y	Y	Y	Y	Y	Y	Y	Y	Y	Y
Student perspectives of extended clinical placements in Optometry										
Qualitative study by Ebrahim et al. (2019):	Y	Y	Y	Y	Y	N	U	Y	Y	Y
Public sector optometrists’ perspective on a decentralised model of clinical training for optometry in KwaZulu-Natal, South Africa										
Qualitative study by Moodley and Singh (2020):	Y	Y	Y	Y	Y	Y	Y	Y	Y	Y
Assessment consolidates undergraduate students’ learning of community-based education										
Qualitative study by Hindi et al. (2022):	Y	Y	Y	Y	Y	Y	Y	Y	Y	Y
Contribution of supervision to the development of advanced practitioners										

Y, yes; N, no; U, unclear.

**Table:**

Category	Study 1	Study 2	Study 3	Study 4
Study design level of evidence	III (C)	III (C)	III (C)	III(C)
Quality Grading	10/10 = 100% ≥ A – High quality	9/10 = B – 90% ≥ A – High quality	10/10 = 100% ≥ A – High quality	10/10 = 100% ≥ A – High quality
Is this a reputable source of evidence?	Yes	Yes	Yes	Yes

### Critical appraisal tool report of eligible *systematic review studies*.

Predetermined questions:

Is the review question/objective clearly and explicitly stated?

Were the inclusion criteria appropriate for the review question?

Was the search strategy appropriate?

Were the sources and resources used to search for studies adequate?

Were the criteria for appraising studies appropriate?

Was critical appraisal conducted by two or more reviewers independently?

Were there methods to minimise errors in data extraction?

Were the methods used to combine studies appropriate?

Was the likelihood of publication bias assessed?

Were recommendations for policy and/or practice supported by the reported data?

Were the specific directives for new research appropriate?

**Table:**

Study type, author, year and title	Q1	Q2	Q3	Q4	Q5	Q6	Q7	Q8	Q9	Q10	Q11
Scoping Review by Dean et al. (2020):	Y	U	Y	Y	Y	Y	Y	Y	N/A	Y	U
Ophthalmology training in sub-Saharan Africa											
Systematic Review by Lee et al. (2020):	Y	Y	Y	Y	Y	Y	Y	Y	N/A	Y	Y
Simulation-based training tools for technical and non-technical skills in ophthalmology											
Systematic Review by Pashmdarfard and Shafaroodi (2018):	Y	Y	Y	Y	Y	Y	Y	Y	N/A	Y	Y
Factors affecting the clinical education of rehabilitation students in Iran											
Systematic Review by Seaman et al. (2022):	Y	Y	Y	Y	Y	Y	Y	Y	N/A	Y	U
Effect of rural clinical placements on intention to practice and employment in rural Australia											

Y, yes; U, unclear; N/A, not applicable.

**Table:**

Category	Study 1	Study 2	Study 3	Study 4
Study design level of evidence	I (A)	I (A)	I (A)	I (A)
Quality Grading	9/10 yes = 90% ≥ A – High quality	10/10 = 100% ≥ A – High quality	10/10 = 100% ≥ A – High quality	9/10 = 90% ≥ A – High quality
Is this a reputable source of evidence?	Yes	Yes	Yes	Yes

## Appendix 2

**TABLE 1-A2:** Summary of data extracted.

No.	Authors, year of study and title	Type of study	Aim and/or objectives	Participants	Results (outcomes, interventions and/or recommendations)
**GOOGLE SCHOLAR**					
1.	Dean et al (2020): **Ophthalmology training in sub-Saharan Africa**	Scoping review	To determine the development of collaborative regional training programmes for ophthalmologists that will improve the training quality and increase number of trainees.	Ophthalmology training documents.	The study found that various strategies were implemented to harmonise the curriculum for ophthalmology training across sub-Saharan Africa. The responsible committee ensured that there is a link of policies/standards/guidelines for facilities and institutions to use and follow in conducting training, fellowship examinations, continuous development trainings, research capacity building as well as mentorship programmes for trainee ophthalmologists. The review found that: National regulators oversee ophthalmology training, examination standards and accreditation. Colleges develop own residency training programme and exam. Districts and hospitals are periodically accredited based on capacity and used as training centres to provide residents with exposure and volume of patients. Speciality placements are scarce because of shortage of specialist ophthalmologists and other staff, in such instances, simulation, dry and wet labs are used for surgery training. Where hospitals do not generate enough patient loads, institutions collaborate with external or international groups to facilitate training of ophthalmologists. Broader collaborations with the pooling of educational resources and joint teaching appointments are used to address shortage of staff. Training the trainer programme equipped the trainers in teaching and cascade knowledge and skills. The transfer and assessment of desirable character, attitude, ethics and responsibilities is yet to be proven. Despite systems in place in various countries, ophthalmology training is complex and multifaceted, and there are still some challenges with respect to capacity and funding of training institutions, including workforce.
2.	Lee et al. (2020): **Simulation-based training tools for technical and non-technical skills in ophthalmology**	Systematic review	To comprehensively evaluate the effectiveness and validity of all simulator models developed for ophthalmology training.	Ophthalmology trainees.	The study found that: Simulations offered a platform for trainees to improve their clinical and surgical skills, as this enables focused, competency-based training without putting patients at risk. It offered potential cost-efficient, maximal skills transfer in minimal time. No retrospective studies were conducted to support that the improved performance is because of simulation. Simulations remove biasness and provide objective assessment of skills level of trainees. The following simulation models were found to be in use, although most studies lacked formal validation processes: - Wet labs for a variety of surgical procedures (anterior and/or posterior segment) on either animal or porcine eyes. - Dry labs to practice diagnostic examination techniques and some posterior segment procedures/surgeries. - E-learning (computer-assisted learning programs and case-based modules) platforms to train technical skills and improve cognitive and other non-technical skills such as leadership and teamwork. Simulation disadvantages included lack of realism, limitations to specific task training rather than comprehensive training and set-up costs.
3.	Pashmdarfard and Shafaroodi (2018): **Factors affecting the clinical education of rehabilitation students in Iran**	Systematic review	To extract the main factors influencing clinical education of rehabilitation science students in Iran.	Rehabilitation science students in Iran.	The study reported evidence of students not able to apply learned theoretical courses in clinical practice, thus revealing a gap between theory and practice. This may be because of: Individual factors (student and trainer characteristics). Environmental factors (ministry policies, educational environment and resources, curriculum structure, amongst others). Clinical factors (trainer experience and competence, clinical environment and resources, different clinical education and supervision). These factors were found to contribute towards student clinical competence, either positively or negatively and towards student professional ethics and motivation. Identification and improvement of the contributory factors early are recommended, in order to overcome obstacles and achieve desirable clinical education with satisfactory levels of student clinical competence.
4.	Seaman et al. (2022): **Effect of rural clinical placements on intention to practice and employment in rural Australia**	Systematic review	To examine non-medicine clinical placement (CP) and rural practice intentions and rural workforce outcomes.	Non-medical professions in Australia undertaking clinical training at a health or other organisational settings.	The study found that: CP of non-medicine health professions at various health settings, including optometry, influence the decision of practitioners to work in those areas. High-quality CP has a positive effect on work intentions/ career employment of students, but there is no evidence on their long-term intentions. The duration of clinical placement per professions differs with no conclusive evidence that duration has an impact upon an area of practice. In some studies, those who stayed more than one year in one area had no intention to work there, and those with less exposure had positive intentions, with opposing findings in other studies. Few studies looked into the intention and attitude of students towards an area of practice prior to CP versus post CP. The high number of students with intention to work in areas they were exposed to, could not be concluded was because of CP. CP can therefore, have both positive and negative effects on student practice location.
5.	Van Vuuren (2017): **Integrated literature review of undergraduate peer teaching in allied health professions**	Literature review	To identify peer-assisted learning (PAL) in allied health profession programmes and the dimensions of PAL.	Undergraduate allied health professions students in South Africa.	The study found that: Knowledge of PAL is limited amongst the allied health professions in South Africa. Only PAL studies in physiotherapy and occupational therapy were included. PAL may address some of the needs of the new generation of students and may be beneficial to the competence of student tutors, tutees and clinical supervisors. PAL has various dimensions that can contribute towards enhancing student clinical knowledge, skills and competence. They include training of tutors and tutees, formality of teaching encounter, evaluation of tutors and tutees, manageable group size and continuous review of outcomes of PAL related to knowledge, skills and attitudes of tutors or tutees.
**EBSCOHOST**					
6.	Chee (2021): **Assessing the use of smartphone app to teach eye screening to opticians**	Experimental study	To evaluate the effectiveness of using video-based application in smartphones to teach community-based eye screening to opticians in comparison to conventional instructor-led lecture and workshop.	Opticians registered with optometrists and opticians board of Singapore.	From the two groups taught on how to conduct Torchlight Eye Screening Test (TEST), a technique used by opticians for community-based screening of patients; where, one group was taught via the conventional classroom face-to-face teaching (F2FT) and another through mobile app teaching (TEACHES-LEM), the study found that: Both methods of teaching were effective with similar knowledge acquisition and retention. E-learning mobile apps, coupled with effective learning strategies can function as an efficient clinical tool, saving man-hour and physical gatherings.
7.	Jonuscheit S. et al (2021): **COVID-19: Ensuring safe clinical teaching at university optometry schools**	Descriptive (opinion) study	To describe the challenges that occurred and continue to affect teaching at optometry schools around the world because of COVID-19 pandemic.	Six optometry offering universities in five countries across four continents (Glasgow-UK; SUNY-US; UCB-US; HKPU-China; UVA-Spain; QUT-Australia).	The study found that: Blended learning and stricter hygiene methods in patient care were introduced to reform optometry education. Optometry schools had to facilitate safe and effective delivery of clinical teaching to reduce the risk of spreading diseases, and where possible, use of alternative methods of examining/testing patients and/or students. Opportunities for changes in optometry education for future consideration include a pooled database of clinical case scenarios for access by collaborating universities; and remote teaching modalities of some clinical skills through guided remote observation/ simulation/ virtual and augmented reality. The changes need proper planning and support of digital learning technologies.
8.	Sehgal et al. (2021): **Impact of COVID-19 on Indian Optometrist: a student, educator and practitioner’s perspective**	Survey	The impact of COVID-19 on optometry education and practices in India.	Optometry educators, students and practitioners in India.	The study found that: Most universities are continuing with online teaching and learning methods post-lockdown. The pandemic reformed optometry education through blended learning methods. Stricter hygiene methods were introduced and are adhered to in patient care. Telemedicine has emerged and adopted as the preferred mode of providing care (examine and diagnose) by most (55%) practitioners in hospitals and private practices.
9.	Kirkman (2022): **Student perspectives of extended clinical placements in Optometry**	Qualitative study	To explore factors influencing placement success and satisfaction from the perspective of optometry students.	Final year optometry students.	The study found factors influencing placement success and satisfaction of students to be: Clear guidance covering training expectations. Support system, to seek help when encountering problems. Opportunity to explore various working environments and meet new people, Opportunity to experience life as a worker and begin thinking of themselves as clinicians. Non-obligation to commit to an employment contract. Independence and the ability to manage challenges encountered both personally and professionally, which equipped them for future practice. Ongoing professional development and pedagogical training for supervisors were recommended, for them to be abreast of new developments.
**SCIENCE DIRECT**					
10.	Schafheutle et al. (2018): **The influence of learning environment on trainee pharmacy technicians’ education and training experience**	Mixed methods study	To capture the views on training experiences of pre-registration trainee pharmacy technicians (PTPT), focusing on differences in community and hospital settings.	Recently registered PTPTs in Great Britain.	The study found that: Trainees from both private (community) and public (hospital) sectors (facilities) were generally satisfied with their experience and they had support from their employing organisations, line managers and colleagues (for hospital trainees). However, those from hospitals enjoyed more benefits than their peers at private settings. Community trainees were mostly alone with no colleagues, only supervisors, reporting that this made them feel isolated without peer support or role models. They had limited exposure to a wide range of activities because of lack of clarity with respect to the roles, responsibilities and accountability of their profession. Hospital trainees showed higher satisfaction scores; they worked in larger teams, consisting of peers, role models, supervisors, support staff and other health professionals, as they were not confined one unit. Clarity on competencies expected upon registration with clear regulatory standards and guidance can ensure that training is structured and delivered in a suitable, equitable and transparent manner. There is a need for policy makers, educators, supervisors and the regulator to research, review and define roles, responsibilities and competencies of PTs.
**SABINET**					
11.	Ebrahim et al (2019): **Public sector optometrists’ perspective on a decentralised model of clinical training for optometry in KwaZulu-Natal, South Africa**	Qualitative study	To explore perspectives of KwaZulu-Natal public sector optometrists on decentralised clinical training in optometry.	Optometrists employed within public sector facilities in KwaZulu-Natal.	The study found that: Public decentralised clinical training (DCT) for optometry students enhances clinical training of optometry students by promoting context-appropriate training and facilitates increased access to eye care services. The training offers learning opportunities for students by exposing them to large patient numbers and a wide range of clinical cases. The challenges in the public sector in the delivery of comprehensive eye health services are lack of resources (equipment and space) appropriate to the required academic training standards for delivery of service, which does not only affect service delivery, but also student clinical training. DCT serves as an advantage for students because there is a high volume of patients in public sector facilities, and students serve as additional human resources so that patients do not need to wait in a queue for long. Slower pace of students and the need to train according to the regulatory standards affect throughput rates in busy public sector clinics. The new environment with high workloads (realities of public sector) compared to the practice environment at university clinics seemed to overwhelm students. Junior optometrists are generally more in touch with theory as compared to senior optometrists but lack comprehensive experience as clinicians and the relevant maturity to serve as clinical teachers for undergraduate optometry students. There is a need for support and mentorship programmes for public sector optometrists to empower both young (junior) and old (senior) optometry clinical trainers in DCT. Development of clinical educator models are required to promote standardised training and practice approaches.
12.	Moodley and Singh (2020): **Assessment consolidates undergraduate students’ learning of community-based education**	Qualitative study	To describe the methods used for assessment of community-based education (CBE) by various disciplines in the School of Health Sciences, [information redacted to maintain the integrity of the review process], South Africa and determine how they align to the anticipated learning outcomes.	Academic leaders of the eight health science disciplines in [information redacted to maintain the integrity of the review process] and one teaching and learning academic leader in the school of health sciences.	The study found that: Clinical assessments in health sciences education optimises student capabilities and protects the public against incompetent clinicians. Assessment ensures that students remain engaged and inculcates the habit of self-reflection, contributing to lifelong learning. Concerns exists over who should conduct assessment as the competence of clinical supervisors varies among programmes, and whether the academics should participate at the CBE sites. Academics opined that part of the assessment responsibility should be shared with clinical supervisors at CBE sites. Workshops or continuous professional development activities should be conducted to empower CBE clinical supervisors on clinical assessment.
**SCOPUS**					
13.	Hindi et al. (2022): **Contribution of supervision to the development of advanced practitioners**	Qualitative study	To apply educational theory in exploring how supervision can contribute to the development of advanced practitioners using the example of several post-registration primary care training pathways for pharmacy professionals (pharmacies and pharmacy technicians).	Advanced pharmacy learners and supervisors.	The study found that the key elements for effective supervision included educational supervision to support learners in identifying their learning needs and provide ongoing support as they progress through a learning pathway; and clinical supervision to guide learners in their everyday work activities, regular pre-arranged face-to-face meetings and ad-hoc contact. Therefore, effective supervision requires: Consistency with regard to availability and accessibility of the supervisor, knowledge and experience and level of support. A flexible approach suited to local circumstances and context of the setting. Learners gaining experience and using skills gained across the workplace from various supervisors, including from other healthcare professions to learn and understand how different professions provide patient care. Support to supervisors through establishing networks where supervisors collaborate, share experiences.

CP, clinical placement; PAL, peer-assisted learning; TEST, Torchlight Eye Screening Test; F2FT, face-to-face teaching; PTPT, pre-registration trainee pharmacy technicians; DCT, decentralised clinical training; CBE, community-based education; TEACHES-LEM, TEACHES-Learning Electronic Module; GCU-UK, Glasgow Caledonian University, United Kingdom; SUNY-USA, State University of New York, United States; UCB-USA, University of California Berkeley, United States; HKPU-China, the Hong Kong Polytechnic University, China; UVA-Spain, University of Valladolid, Spain; QUT-Australia, Queensland University of Technology, Australia.
